# Supplementary material for: Circular economy, environmental quality and tourism receipts in Europe: A time series data analysis
Source: PLoS One. 2023 Nov 30;18(11):e0288098. doi: 10.1371/journal.pone.0288098 (PMC10688725; doi:10.1371/journal.pone.0288098)
Supplement: S1 File — (DOCX) [file pone.0288098.s003.docx]

**[Investigating the influence of the circular economy on tourism receipts: Evidence from panel analysis of the European Union](https://www.sciencedirect.com/science/article/pii/S0261517713000678?casa_token=s4VY9PCa0uMAAAAA:LyAIbTNdhfwb1FFrid0Y8NwcLpx46AHe45QrG0Tu_MzHYG3rzkT8pmy14kMHXZwxhcuN_w18)**

**CRediT Author Statement**

**Dr Michael Odei Erdiaw-Kwasie –** Conceptualization, Roles/Writing - original draft; Writing - review & editing, Supervision

**Mr. Kofi Kusi Owusu-Ansah** – Methodology; Data Curation

**Dr Matthew Abunyewah -** Roles/Writing - original draft; Writing - review & editing.

**Professor Khorshed Alam –** Supervision; Writing - review & editing

**Mr Patrick Arhin -** Roles/Writing - original draft; Writing - review & editing
