# Supplementary material for: Circular economy, environmental quality and tourism receipts in Europe: A time series data analysis
Source: PLoS One. 2023 Nov 30;18(11):e0288098. doi: 10.1371/journal.pone.0288098 (PMC10688725; doi:10.1371/journal.pone.0288098)
Supplement: Attachment — Submitted filename: review.docx [file pone.0288098.s004.docx]

Please note the following comments:

My comments relate mostly to the methodology of the study

Is it true that there are only 17 observations for the long run models for Denmark and Finland in Table 5? This is too less, even for ARDL models. Also, it is surprising that only 2 of the many countries considered are cointegrated. I find this very hard to believe. Because the data span is so less for each country, why not consider panel ARDL models such as the pooled mean group approach which is also developed by Pesaran, Shin and Smith (1999).

The results for Denmark and Finland are stable according to the CUSUM and CUSUMSQ plots. However, many of the other countries are not. This instability can possibly impact your long run results affecting the outcome of cointegration. It may indicate specification issues such as an incorrect functional form or the exclusion of structural breaks, however I do not know how you can possibly justify including structural breaks with a sample size of just 17! I then went to check the results of the RESET test in Table 2 and to my surprise, the notes to this table indicate that p-values are reported in parenthesis. I did not know that p-values could go up to 5.86. Please check before submitting.

Therefore, my recommendation is a major revision but the authors need to seriously address the methodological shortcomings of their paper.
