# Supplementary material for: Circular economy, environmental quality and tourism receipts in Europe: A time series data analysis
Source: PLoS One. 2023 Nov 30;18(11):e0288098. doi: 10.1371/journal.pone.0288098 (PMC10688725; doi:10.1371/journal.pone.0288098)
Supplement: Attachment — Submitted filename: Authors Response_REVISED.docx [file pone.0288098.s005.docx]

| **REVIEWERS COMMENTS** | **AUTHORS RESPONSE** |
| --- | --- |
| **ASSISTANT EDITOR** | |
| 1. Please ensure that your manuscript meets PLOS ONE's style requirements, including those for file naming. The PLOS ONE style templates can be found at | We have restructured the manuscript in line with the requirement of the journal. Also, the file naming has been revised accordingly. |
| . Thank you for stating the following financial disclosure:  "No funding"  At this time, please address the following queries:  a) Please clarify the sources of funding (financial or material support) for your study. List the grants or organizations that supported your study, including funding received from your institution.  b) State what role the funders took in the study. If the funders had no role in your study, please state: “The funders had no role in study design, data collection and analysis, decision to publish, or preparation of the manuscript.” c) If any authors received a salary from any of your funders, please state which authors and which funders. d) If you did not receive any funding for this study, please state: “The authors received no specific funding for this work.”  Please include your amended statements within your cover letter; we will change the online submission form on your behalf. | The authors have reviewed the funding section and confirm that all details are correct.  The authors have included a section in the cover letter indicating that the authors received no specific funding for this work. |
| 3. Thank you for stating the following in your Competing Interests section:   "No competing interest"  Please complete your Competing Interests on the online submission form to state any Competing Interests. If you have no competing interests, please state "The authors have declared that no competing interests exist.", as detailed online in our guide for authors at <http://journals.plos.org/plosone/s/submit-now>   This information should be included in your cover letter; we will change the online submission form on your behalf. | The authors have completed the competing interest section in the online portal as directed. The authors have included a section in the cover letter indicating that the authors have no competing interest. |
| Please note that in order to use the direct billing option the corresponding author must be affiliated with the chosen institute. Please either amend your manuscript to change the affiliation or corresponding author, or email us at [plosone@plos.org](mailto:plosone@plos.org) with a request to remove this option. | The authors have noted the details and confirm that our institution has no direct billing agreement with Plosone. The payment will be made by the corresponding author |
| We note that you have indicated that data from this study are available upon request. PLOS only allows data to be available upon request if there are legal or ethical restrictions on sharing data publicly. For more information on unacceptable data access restrictions, please see <http://journals.plos.org/plosone/s/data-availability#loc-unacceptable-data-access-restrictions>  In your revised cover letter, please address the following prompts:  a) If there are ethical or legal restrictions on sharing a de-identified data set, please explain them in detail (e.g., data contain potentially sensitive information, data are owned by a third-party organization, etc.) and who has imposed them (e.g., an ethics committee). Please also provide contact information for a data access committee, ethics committee, or other institutional body to which data requests may be sent.  b) If there are no restrictions, please upload the minimal anonymized data set necessary to replicate your study findings as either Supporting Information files or to a stable, public repository and provide us with the relevant URLs, DOIs, or accession numbers. For a list of acceptable repositories, please see <http://journals.plos.org/plosone/s/data-availability#loc-recommended-repositories>.  We will update your Data Availability statement on your behalf to reflect the information you provide. | We have revised our cover letter accordingly and confirm there is no restrictions for the data used for this study. As part of the upload, the authors have uploaded the minimal anonymized data set necessary to replicate your study findings as Supporting Information files. |
| 6. Please ensure that you include a title page within your main document. We do appreciate that you have a title page document uploaded as a separate file, however, as per our author guidelines (<http://journals.plos.org/plosone/s/submission-guidelines#loc-title-page>) we do require this to be part of the manuscript file itself and not uploaded separately. Could you therefore please include the title page into the beginning of your manuscript file itself, listing all authors and affiliations? | The authors have included the title page to the main manuscript |
| **REVIEWER 1** | |
| 1. Sustainability and Circular Economy – There can be a discussion on this included in the Literature Review as circular economy practices may be deemed very similar to sustainable practices by readers. Therefore, a clear distinction needs to be made and the connection, for example, whether sustainable practices drive circular economy or vice-versa. | We thank the reviewer. The authors have introduced a section in the literature review that offers the distinction between sustainability and circular economy and how each concept drives the other.  [Refer to section 2.2, p.6] |
| 2. Page 5 – Stern et al, 1999 – capital “S” | We have addressed the comment. |
| 3. Font colour – Black (In certain places in seems that text is grey | The entire manuscript has been edited and all grey sections edited to black. |
| 4. Manuscript structure - align to that of the journal. | We thank the reviewer. We have revised the manuscript to align with the journal’s structure. |
| 5. CUSUM squared issue for Germany and Romania. | The authors have revised the earlier reported CUSUM figures as the authors re-run the data to ensure that all errors identified by reviewers are addressed. The figures for Germany and Romania have been revised. |
| 6. Include limitations for the study. | The authors have included a section on the limitation of the study.  [Refer to section 6, page 17] |
| 7. Use Vancouver Style for referencing | The authors have revised the references in line with the Vancouver style |
| **REVIEWER 2** | |
| I would like to sincerely thank the authors for submitting the article entitled: Circular Economy and tourism receipts in Europe: A Panel data analysis. This study is exciting, and the authors have managed to gather insights using panel data and contribute towards the tourism literature. While the results are noteworthy there are limitations of this paper that may not meet the novelty of the journal currently. The following critics will be beneficial in reshaping the paper suitable for the journal in future | We thank the reviewer for the comments. The authors have gone through all the comments and addressed each step by step. |
| 1. I suggest the authors read the instructions for formatting the paper as per the requirement of the journal. (Font colour varies in certain places and use of citation) | The authors have thoroughly revised the paper in accordance with the journal’s requirements. We have also formatted the font colour to ensure all grey sections are changed to black. |
| 2. Justify your reasoning for employing the ARDL approach from tourism literature and perspective. | The authors have clearly highlighted the justification for adopting the ARDL model for this study.  [Refer to section 3.1, p.8] |
| 3. The "Value-Belief-Norm of environmentalism" theory lacks supporting evidence from country-specific studies. Provide literature specific to the model proposed and its relevance to tourism in some countries. | The authors have provided country-specific case studies using the VBN of environmentalism" theory.  [Refer to section 2.1, para 2] |
| 4. The second theoretical model incorporated is the destination image model. Please explore this angle further and complement the research contribution. | We thank the reviewer for the comment. The authors have introduced new sections that capture more details on the importance of the destination image model.  [Refer to section 2.1, para 2, 3] |
| 5. There is an overall gap between research findings and the linkage towards the paper's uniqueness. | The authors have strengthened the link between the gap and the research findings. First, the authors have rewritten the section on the gap to enhance clarity. Adding to that, we have linked the identified gap to the contribution of the study. The authors have rewritten the contribution section to enhance clarity.  [Refer to section 1] |
| 6. Please be specific to which SDG Goal the present study is related; (egSDG1, 2, 3 ….), and highlight the particular goal in the abstract in some parts of the literature. | The authors have rewritten some sections of the literature to explicitly indicate which of the SDGs can be impacted by circular models. We have also linked it to the ongoing argument levelled in the paper.  [Refer to section 2.2, pp. 7-8] |
| 7.  Provide a citation for the statement that claims "Consequently, such an image can increase tourism receipts and increase the number of tourists. However, although it is great to have more tourists unless these tourists are eco-tourists and careful about their consumption, the influx of tourists can be even worse for the environment."( Page 6) | The authors have revisited the section, revised the content and duly cited relevant articles.  [Refer to section 2.1, para. 3] |
| 8. Include a separate paragraph on Circular economy and Sustainable tourism. | We thank the reviewer. The authors have introduced a new paragraph in the literature review section that provides insights into the relationship between circular economy and sustainable tourism. All relevant articles on the topic have been duly cited by the authors.  [Refer to section 2.2, para. 3] |
| 9. Shorten long sentence into one sentence or split the sentence:  " Furthermore, the increasing public awareness that climate change and other severe environmental problems must be addressed through change practices indicates that a growing segment of tourists actively searches for destinations, accommodations, and holiday experiences with a clear sustainability profile (Buffa, 2015; Doran et al., 2017), although some studies indicate that environmental concerns translate stronger into behaviour at home than on vacation (Mehmetoglu, 2010)." | The authors have revised the section to enhance readability. The sentence has been shortened as suggested by the reviewer. |
| 10. Page 7, second paragraph, first line ; please rephrase with a meaningful outcome as it is unclear. "and the linear consumption model endemic to the tourism industry, literature on circular economy and tourism receipts has predominantly focused on how the former may assist with the many challenges in the sector (Manniche et al., 2017)." Please rectify the phrase "how the former may assist with the many challenges in the sector" it is a clear indication of the passive sentence. Also, what does "the former" mean in the sentence? | The authors have revised the section. More specifically, the authors have rewritten the section to enhance readability. |
| 11. The methodology section is well articulated | We thank the reviewer |
| 12. How is the circular-led tourism hypothesis justified in the discussion section? Where has this been used previously? | The authors have revisited the section and made the necessary changes. We have deleted the sentence as it appears to break the flow of the argument presented in that paragraph. Also, the authors have rewritten some sections of the paragraph to enhance readability. |
| 13. It is suggested that discussion of results is well suited with theoretical consideration. Since there are two models being proposed in the literature but nothing has been mentioned in the discussion. I suggest linking the model being proposed in the literature and supporting with originality of this paper. | We thank the reviewer for such an important comment. The reviewers have introduced a new section in the discussion that draws from the theoretical evidence and study findings to offer direction for linking the two models guiding this study. The section now reads:  “” |
| 14. Please be mindful of the policy recommendation in the conclusion section. Justify your policy and its relevance to particular sectors. | We thank the reviewer for the comment. The authors have introduced a new paragraph to address the policy section of the study. The section now reads:  “The findings of this study have important policy implications. Circular economy increases tourism receipts in Europe's top tourism destination, according to the empirical findings. Tourism-led countries must urgently promote circular initiatives that are regenerative in nature in order to counter the impact of unsustainable tourism activities on the environment. To effectively deal with unsustainable tourism's degrading effects, policymakers need to implement and monitor a strict environmental framework. Also, the circular economy promotes environmental quality, and good ecological footprints lead to higher tourism receipts because they attract a large number of tourists. There is therefore a need for tourism-led countries to shift from over reliant on environmentally damaging practices like single plastics, virgin materials, and increased waste generation to investing in circular economy initiatives like promoting reusable materials, recycling tourist waste, and utilizing secondary raw materials. By reducing environmental problems associated with unsustainable tourism across a wide range of tourist destinations, circular economy initiatives can help improve their overall revenue. In order to achieve environmental sustainability in the tourism sector, policymakers should use circular economy standards as part of the regulatory framework.” |
| 15. Attached are the following works of literature that will be useful in replenishing the current study (a) Hernández, J. M., & León, C. J. (2013). Welfare and Environmental Degradation in a Tourism-Based Economy. Tourism Economics, 19(1), 5–35. <https://doi.org/10.5367/te.2013.0191> (b) Kumar, N. N., Patel, A., Chandra, R. A., & Kumar, N. N. (2021). Publication bias and the tourism-led growth hypothesis. PloS one, 16(10), e0258730 | We thank the reviewer for the suggestions. The authors have read all suggested articles, and all have been duly cited in the manuscript. |
| **REVIEWER 3** | |
| My comments relate mostly to the methodology of the study  Is it true that there are only 17 observations for the long run models for Denmark and Finland in Table 5? This is too less, even for ARDL models. Also, it is surprising that only 2 of the many countries considered are cointegrated. I find this very hard to believe. Because the data span is so less for each country, why not consider panel ARDL models such as the pooled mean group approach which is also developed by Pesaran, Shin and Smith (1999). | We thank the reviewer for the helpful comments. The authors have significantly revised the methodology section to address the concerns.  The authors have run a short-run analysis using an actual error correction model rather than the ARDL model to overcome the earlier identified weaknesses identified by the reviewer.  In addition to the ARDL model used to estimate the long-run relationship between the variables, the authors have confirmed running the ECM model followed by the error correction term, which captures the short run dynamics of the variables to overcome the earlier identified weaknesses by the reviewer.  . |
| The results for Denmark and Finland are stable according to the CUSUM and CUSUMSQ plots. However, many of the other countries are not. This instability can possibly impact your long run results affecting the outcome of cointegration. It may indicate specification issues such as an incorrect functional form or the exclusion of structural breaks, however I do not know how you can possibly justify including structural breaks with a sample size of just 17! I then went to check the results of the RESET test in Table 2 and to my surprise, the notes to this table indicate that p-values are reported in parenthesis. I did not know that p-values could go up to 5.86. Please check before submitting. | Also, the authors identified and corrected a mistake regarding the P-values; the values shown were the actual figures rather than those in brackets. We re-ran the diagnostic tests, converting all diagnostic test values to P-values. The outcome has helped address the comment about the P-value raised by the reviewer.  Additionally, the authors have incorporated the ECT term into the short-run narrative, which reflects the speed of adjustment toward long-run equilibrium when there is a deviation from the long-run equilibrium. This was particularly relevant for Denmark and Finland, which showed a long-run relationship. |
| Therefore, my recommendation is a major revision, but the authors need to seriously address the methodological shortcomings of their paper. | The authors have considered all the reviewer’s concerns and re-run several new tests to overcome the identified weaknesses in the methodology section of the paper. |
