# Supplementary material for: Circular economy, environmental quality and tourism receipts in Europe: A time series data analysis
Source: PLoS One. 2023 Nov 30;18(11):e0288098. doi: 10.1371/journal.pone.0288098 (PMC10688725; doi:10.1371/journal.pone.0288098)
Supplement: Attachment — Submitted filename: Authors Response_REVISED.docx [file pone.0288098.s006.docx]

| **REVIEWERS COMMENTS** | **AUTHORS RESPONSE** |
| --- | --- |
| **EDITOR** | |
| Thank you for submitting your manuscript to PLOS ONE. After careful consideration, we feel that it has merit but does not fully meet PLOS ONE’s publication criteria as it currently stands. Therefore, we invite you to submit a revised version of the manuscript that addresses the points raised during the review process. | We thank the Editor. The authors have read all the reviewers’ comments and addressed them accordingly. |
| **REVIEWERS** | |
| 1. If the authors have adequately addressed your comments raised in a previous round of review and you feel that this manuscript is now acceptable for publication, you may indicate that here to bypass the “Comments to the Author” section, enter your conflict of interest statement in the “Confidential to Editor” section, and submit your "Accept" recommendation.  Reviewer #1: All comments have been addressed  Reviewer #2: All comments have been addressed. | We thank the reviewers. |
| 2. Is the manuscript technically sound, and do the data support the conclusions?  The manuscript must describe a technically sound piece of scientific research with data that supports the conclusions. Experiments must have been conducted rigorously, with appropriate controls, replication, and sample sizes. The conclusions must be drawn appropriately based on the data presented.  Reviewer #1: Partly  Reviewer #2: Yes | We thank the reviewers. The authors have re-read the section to improve readability. |
| 3. Has the statistical analysis been performed appropriately and rigorously?  Reviewer #1: I Don't Know  Reviewer #2: Yes | We thank the reviewers |
| 4. Have the authors made all data underlying the findings in their manuscript fully available?  The [PLOS Data policy](https://aus01.safelinks.protection.outlook.com/?url=http%3A%2F%2Fwww.plosone.org%2Fstatic%2Fpolicies.action%23sharing&data=05%7C01%7Cmichael.erdiaw-kwasie%40cdu.edu.au%7C3bf3d98b983b4e07670608db6c70c662%7C9f2487678e1a42f3836fc092ab95ff70%7C0%7C0%7C638223003851085214%7CUnknown%7CTWFpbGZsb3d8eyJWIjoiMC4wLjAwMDAiLCJQIjoiV2luMzIiLCJBTiI6Ik1haWwiLCJXVCI6Mn0%3D%7C3000%7C%7C%7C&sdata=pOGwo%2FYy6FIm61FvriuXaBSxH8E%2FjeNLaauOJ%2FcaOFM%3D&reserved=0) requires authors to make all data underlying the findings described in their manuscript fully available without restriction, with rare exception (please refer to the Data Availability Statement in the manuscript PDF file). The data should be provided as part of the manuscript or its supporting information, or deposited to a public repository. For example, in addition to summary statistics, the data points behind means, medians and variance measures should be available. If there are restrictions on publicly sharing data—e.g. participant privacy or use of data from a third party—those must be specified.  Reviewer #1: Yes  Reviewer #2: Yes | We thank the reviewer. |
| 5. Is the manuscript presented in an intelligible fashion and written in standard English?  PLOS ONE does not copyedit accepted manuscripts, so the language in submitted articles must be clear, correct, and unambiguous. Any typographical or grammatical errors should be corrected at revision, so please note any specific errors here.  Reviewer #1: Yes  Reviewer #2: Yes | We thank the reviewers |
| 6. Review Comments to the Author  Please use the space provided to explain your answers to the questions above. You may also include additional comments for the author, including concerns about dual publication, research ethics, or publication ethics. (Please upload your review as an attachment if it exceeds 20,000 characters)  Reviewer #1: All comments have been addressed. However there are some additional coents based on the new version: - on Page 17, the last paragrapgh start with although but there is no indication of although what. Please revise. - On Page 4, 5th line. "patent patents". Please revise or rebut.  Reviewer #2: I would like to thank the authors for responding well towards the comments. I also hope that those comments will be beneficial in future prospects. | The authors have addressed the comments raised by reviewer 1.  [Refer to page 4 and 17] |
| 7. PLOS authors have the option to publish the peer review history of their article ([what does this mean?](https://aus01.safelinks.protection.outlook.com/?url=https%3A%2F%2Fjournals.plos.org%2Fplosone%2Fs%2Feditorial-and-peer-review-process%23loc-peer-review-history&data=05%7C01%7Cmichael.erdiaw-kwasie%40cdu.edu.au%7C3bf3d98b983b4e07670608db6c70c662%7C9f2487678e1a42f3836fc092ab95ff70%7C0%7C0%7C638223003851085214%7CUnknown%7CTWFpbGZsb3d8eyJWIjoiMC4wLjAwMDAiLCJQIjoiV2luMzIiLCJBTiI6Ik1haWwiLCJXVCI6Mn0%3D%7C3000%7C%7C%7C&sdata=H3mmVXPGNCbPaBwT6W51aC%2Fe53YZ511QH750F5BQE3Y%3D&reserved=0)). If published, this will include your full peer review and any attached files.   If you choose “no”, your identity will remain anonymous but your review may still be made public.   **Do you want your identity to be public for this peer review?** For information about this choice, including consent withdrawal, please see our [Privacy Policy](https://aus01.safelinks.protection.outlook.com/?url=https%3A%2F%2Fwww.plos.org%2Fprivacy-policy&data=05%7C01%7Cmichael.erdiaw-kwasie%40cdu.edu.au%7C3bf3d98b983b4e07670608db6c70c662%7C9f2487678e1a42f3836fc092ab95ff70%7C0%7C0%7C638223003851085214%7CUnknown%7CTWFpbGZsb3d8eyJWIjoiMC4wLjAwMDAiLCJQIjoiV2luMzIiLCJBTiI6Ik1haWwiLCJXVCI6Mn0%3D%7C3000%7C%7C%7C&sdata=mDA4HNeWCfyd5k8PNtv5xQmVOOWYLKafO6qoWBNRlzE%3D&reserved=0).  Reviewer #1: No  Reviewer #2: No | N/A |
